# Supplementary material for: Co-Delivery of the Human NY-ESO-1 Tumor-Associated Antigen and Alpha-GalactosylCeramide by Filamentous Bacteriophages Strongly Enhances the Expansion of Tumor-Specific CD8+ T Cells
Source: Viruses. 2023 Mar 2;15(3):672. doi: 10.3390/v15030672 (PMC10059692; doi:10.3390/v15030672)
Supplement: Supplementary file 1 [file viruses-15-00672-s001.zip › viruses-2139526-supplementary.pdf]

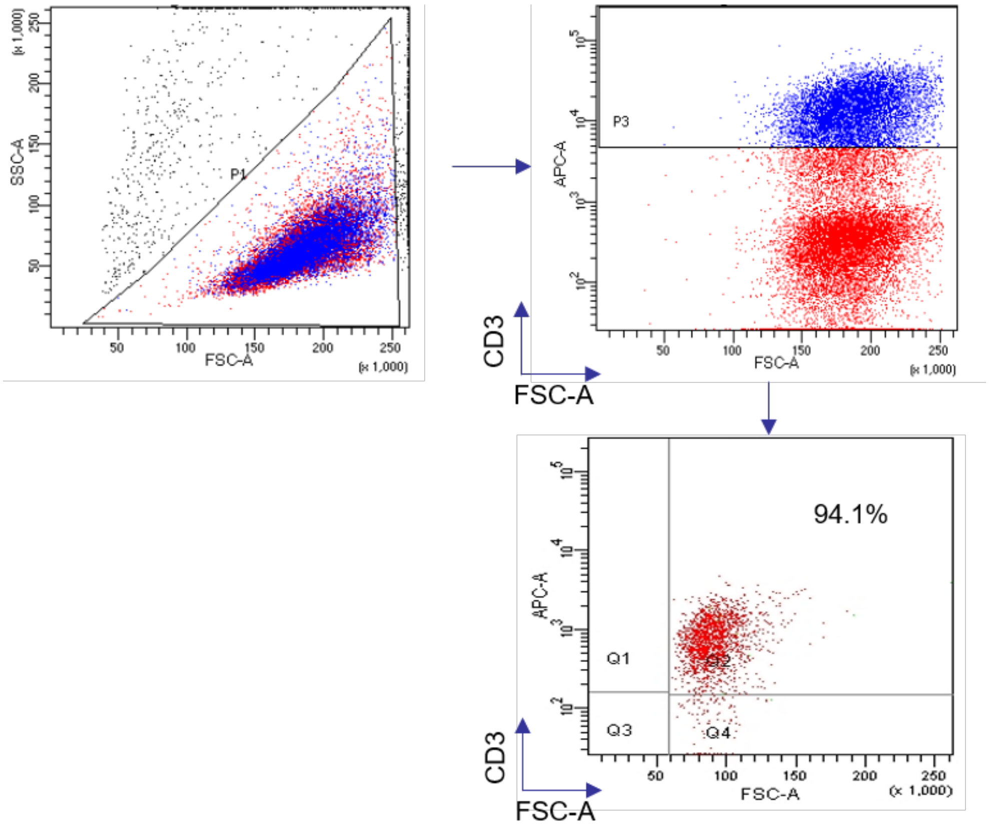

Figure S1. J76 NFAT-GFP 1G4 cell sorting. J76 NFAT-GFP cells were transduced with recombinant lentiviral particles encoding the NY-ESO-1 specific 1G4 TCR. 48 hours later, cells were stained with APC-conjugated anti-human CD3 and acquired by FACS. Cells with brighter CD3 signals (P3, blue) were gated and sorted. Sorted cells were then assayed for CD3 expression.

A

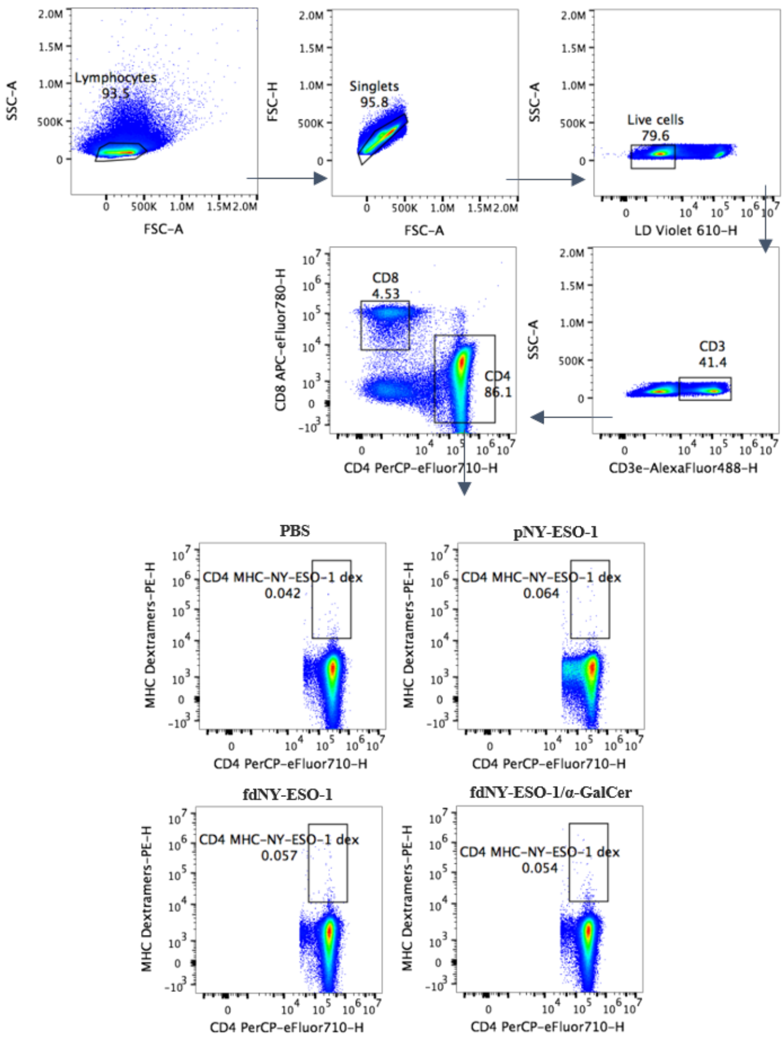

B

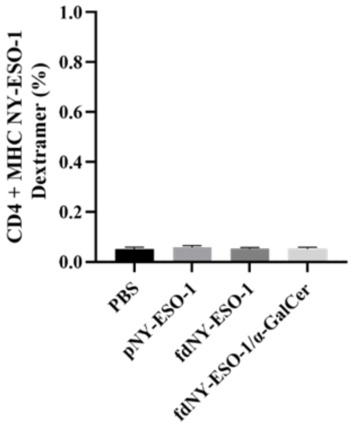

Figure S2. Analysis of MHC-I-NY-ESO-1-dextramers positive on CD4+ T cells (**A**) Gating strategy of one representative sample. Lymphocytes and singlets were gated by dimension; then live cells were gated as negative for live-dead dye. Then CD3+ T cells were gated on live cells and MHC-I NY-ESO-1-dextramer positive cells were shown as percentage on CD4+ gated T cells. (**B**) Percentages of MHC-I NY-ESO-1-dextramer+/CD4+ cells. Mean ± SEM of 4 mice per group are shown.
